# A pilot study of a new app based on self-compassion for the prevention and promotion of mental health among Brazilian college students

**DOI:** 10.3389/fpsyg.2024.1414948

**Published:** 2024-08-23

**Authors:** Bruno Luis Schaab, Lara Finkler Cunha, Desiree Cordoni Silveira, Pamela Carvalho da Silva, Kellen Greff Ballejos, Gabriela Bertoletti Diaz, Vanessa Kaiser, Prisla Ücker Calvetti, Sílvio César Cazella, Helena Maria Tannhauser Barros, Caroline Tozzi Reppold

**Affiliations:** ^1^Psychological Assessment Laboratory, Health Sciences Program, Federal University of Health Sciences of Porto Alegre (UFCSPA), Porto Alegre, Brazil; ^2^Department of Exact Sciences and Social Applied Science, Health Sciences Program, Federal University of Health Sciences of Porto Alegre (UFCSPA), Porto Alegre, Brazil; ^3^Psychological Assessment Laboratory, Rehabilitation Sciences Program, Federal University of Health Sciences of Porto Alegre (UFCSPA), Porto Alegre, Brazil; ^4^Department of Pharmacosciences, Health Sciences Program, Federal University of Health Sciences of Porto Alegre (UFCSPA), Porto Alegre, Brazil

**Keywords:** positive psychology, self-compassion, app, students, mental health, intervention

## Abstract

**Introduction:**

Epidemiological data suggest substantial issues on the mental health of university students worldwide. Self-compassion is associated with lower rates of psychological distress and better positive mental health. Thus, we have developed a app-based intervention based on self-compassion principles targeting the prevention and promotion of mental health in college students. The current pilot study assessed adherence to intervention, preliminary mental health benefits, and satisfaction and acceptability with the app among Brazilian college students.

**Methods:**

The study employed a pre-experimental single-group design along with pre-test and post-test assessments (*n* = 23). A mixed methods approach was utilized to comprehensively assess the outcomes of the intervention.

**Results:**

The overall adherence rate for the intervention was 37.87%, with 26.26% of participants successfully completing all modules. Among the 21 outcomes assessed, 15 exhibited statistically significant results. Notably, there was a substantial increase in self-compassion, demonstrating a large effect size (*d* = 1.15), and a moderate effect size reduction in stress (*d* = 0.62) and anxiety (*d* = 0.52). All satisfaction indicators for the intervention received scores above 7. The intervention was well-received by participants who completed it, although a potential barrier identified was the volume of text within the app and the need to adopt a more playful and concise format for the intervention.

**Discussion:**

Despite a notable participant dropout, the adherence to intervention aligns with patterns observed in other online interventions conducted in real-world settings. The observed mental health benefits, high satisfaction levels, and positive acceptance underscore the rationale for pursuing a subsequent randomized clinical trial.

## 1 Introduction

In developed countries and some developing countries, such as Brazil, university life typically spans the period between the end of adolescence and early adulthood. Emotional conflicts typical of this developmental phase, along with the demanding and competitive academic environment, are associated with the onset of mental health disorders and psychopathological symptoms before the professional life of adults (Pedrelli et al., 2019; Broglia et al., 2021; Campbell et al., 2022). Therefore, even before COVID-19, mental health issues of college students were a concern in both educational and health segments. Epidemiological data prior to COVID-19 suggested that up to 35% of college students met criteria for a common mental disorder (Auerbach et al., 2018). In Brazil, between 34 and 49% of college students reported experiencing emotional distress (Graner and Cerqueira, 2019). Since the onset of the COVID-19 pandemic and the necessity for lockdowns, there has been a decline in the mental health status of university students worldwide (Buizza et al., 2022; Lemyre et al., 2023).

Nowadays, even with the end of the social isolation and the return to normal life globally, recent estimates still suggest a high prevalence of mental disorders and psychopathology symptoms among college students, especially depression, anxiety, and stress (Estrada-Araoz et al., 2023; Farfán-Latorre et al., 2023; Liverpool et al., 2023; Dave et al., 2024). These mental health issues are not just a personal burden; they also contribute to several challenges in daily life, such as poor academic performance, impairments in academic adherence (Mboya et al., 2020), difficulties in peers relationships (Dachew et al., 2015), substance use (Mboya et al., 2020), self-harm, risky sexual behaviors, and negative impacts on physical health. In addition, suicide is a significant concern, ranking as the fourth leading cause of death among individuals aged 15 to 29 (World Health Organization, 2019).

Given this, there is a recognized urgency for worldwide policies addressing the mental health of university students, especially for those students from low-income countries or without regular access to mental health care, as in Brazil. Beyond pharmacological interventions, it is crucial the development and offering of programs for fostering psychological resources (Worsley et al., 2022). These psychological programs may contribute to positive mental health and alleviate psychological distress through cultivating emotion regulation skills, that is, the capacity to intentionally manage and control emotions occurrence, intensity and duration through attitudes, behaviors, and thoughts (Leahy et al., 2011).

In recent years, typical dimensions of Positive Psychology (PP) have emerged as promising strategies for emotion regulation, notably self-compassion skills (Inwood and Ferrari, 2018). Self-compassion encompasses attitudes of kindness, tolerance, and support towards oneself during challenging times or when confronted with feelings of failure and inadequacy (Neff, 2023). Also, self-compassion entails recognizing our own suffering with the intention of healing it (Neff, 2011). This involves directing towards ourselves the same compassion that we might extend to a person experiencing distress, pain or facing life challenges (Neff and Germer, 2018; Neff, 2023).

According to seminal author Neff (2023), self-compassion encompasses three interrelated components: self-kindness versus self-judgment, which involves being kind and understanding towards oneself during moments of inadequacy, suffering, or failure instead of being self-critical or neglecting one’s suffering; common humanity versus isolation, which involves recognizing that mistakes and failures are part of the human condition and that we should not feel isolated; and mindfulness versus over-identification, which involves seeing oneself in a balanced and realistic way, without suppressing or exaggerating one’s thoughts or emotions, rather than over-identifying with them (Neff, 2011; Neff and Germer, 2018). Evidence suggests that higher rates of psychological distress among undergraduate students are associated with higher rates of self-criticism (McIntyre et al., 2018) and loneliness (Yang et al., 2023). Self-compassion interventions may cultivate skills that allow individuals to treat themselves with more kindness and less self-criticism; feel less lonely and more connected to others; and recognize their own thoughts and emotions and being mindfulness.

Cross-sectional studies have consistently indicated an association between self-compassion with lower rates of distress and higher rates of positive mental health, including optimism (Zhao et al., 2022), positive affect and life satisfaction (Stoeber et al., 2020), hope (Yang et al., 2016), compassion (López et al., 2018) and emotion regulation (Rech et al., 2023). Self-compassion appears to be particularly effective in alleviating the impacts of self-criticism and self-demand among university students (Wakelin et al., 2022), which are typically linked to poorer mental health (McIntyre et al., 2018). Since self-compassion skills may be learned and applied in everyday life, it’s been suggested to develop and implement self-compassion programs for promoting emotional well-being among university students and young people (Egan et al., 2022; Neff, 2023).

Different therapeutic approaches have focused on cultivating self-compassionate skills, including Compassion Focused Therapy (CFT) (Gilbert, 2010) and Mindful Self-Compassion (MSC) program (Neff and Germer, 2018). Despite specific differences in techniques, format and length of interventions, all these approaches have in common the promotion of self-compassionate skills. Systematic reviews of randomized clinical trials with meta-analysis suggests the efficacy of self-compassion interventions in enhancing self-compassion and, consequently, reducing depression, anxiety, and stress among the general population with moderate effect size (Kirby et al., 2017; Ferrari et al., 2019).

Despite the efficacy of these interventions, the most of them are still provided in a face-to-face format, potentially limiting access to their benefits. Recently, some studies have aimed to adapt these interventions into the online format to university students (Andersson et al., 2021; Serlachius et al., 2021; Beaumont et al., 2022), circumventing barriers present in face-to-face contexts, such as professional costs, service location, privacy and anonymity. The purpose of this app is to cultivate self-compassion skills among Brazilian university students for use in university life, which can reduce their psychological distress and improve positive mental health.

To the best of our knowledge, no digital intervention based on self-compassion specifically targeting Brazilian college students has been previously developed. Furthermore, only one app in Portuguese was found for cultivating self-compassion, which is the app 29K FJN from Portugal. Consequently, we created a new app called “Eu + Compassivo” (translated in English as “Me More Compassionate”) featuring a self-compassion-based intervention delivered through a smartphone app to prevent issues and promote better mental health of Brazilian college students. Thus, the purpose of this new app is to cultivate self-compassion skills among Brazilian university students for use in university life, which can reduce their psychological distress and improve positive mental health. Since this is a population with a high prevalence of common mental disorders, it is believed that college students can benefit from a self-compassion intervention.

As a pilot study, the research aims to identify indicators for conducting a randomized clinical trial (Lancaster, 2015; Lowe, 2019). The objectives include assessing the adherence to app and its preliminary benefits on mental health, along with the assessment of satisfaction and acceptability of the intervention.

## 2 Materials and methods

### 2.1 Eu + Compassivo app

The app constitutes an asynchronous psychoeducational intervention designed for the prevention and promotion of mental health among Brazilian college students. The content of the app primarily draws upon the principles of self-compassion as proposed by Neff (2011), as well as on the literature concerning the mental health of college students (Mofatteh, 2021).

The first step of app development involved drafting an intervention protocol in a PDF format. This protocol comprehensively outlined all facets of the app, including textual elements and psychological exercises aimed at cultivating self-compassion. Subsequently, the intervention protocol was assessed by six psychologists specialized in Positive Psychology and Clinical Psychology, who assessed the suitability of its contents and active components. Following this phase, the app’s coding and design processes commenced, culminating in the creation of version 1.0 of the app (Schaab et al., 2024).

The app comprises eight thematic modules that must be completed within a maximum 45-day timeframe. These modules consist of texts, audios, and videos that connect the principles of self-compassion with university life. Each module also includes exercises within the app to foster self-compassion, encouraging students to integrate these practices into their daily lives. The exercises encompass diverse tasks, such as writing and meditation, and focus on the dimensions outlined in the Self-Compassion Scale — specifically, self-kindness, self-judgment, common humanity, isolation, mindfulness, and over-identification (Neff, 2016). Table 1 presents the structure of the intervention. Below is a summary of each of the eight modules:

**Table 1:** Summary of app modules.

Modules	Theoretical dimension	Self-compassion exercise	Module methodology
1	Welcome to user and introduction to using the Eu + Compassivo app. Mental health and university life – emotion regulation and self-compassion.	Psychoeducation	One psychoeducation text. One psychoeducation video.
2	Self-judgment	Psychoeducation. Compassionate letter. How would you treat a friend?	One psychoeducation text. One psychoeducation video. Two self-compassion exercises involving writing.
3	Self-kindness	Psychoeducation. Imagination of the compassionate self. Self-Compassion Mantra.	One psychoeducation text. One self-compassion exercises involving writing. One self-compassion exercise involving listening to an audio.
4	Mindfulness	Psychoeducation. Compassionate body scanning. Mindfulness exercise (breathing).	One psychoeducation text. Two self-compassion exercise involving listening to an audio.
5	Common humanity	Psychoeducation. People who also have these emotions. Seeing yourself as you are.	One psychoeducation text. Two self-compassion exercises involving writing.
6	Isolation	Psychoeducation. Self-criticism x self-kindness. Self-compassionate pause.	One psychoeducation text. Two self-compassion exercises involving writing.
7	Over-identification	Psychoeducation. Loving kindness meditation.	One psychoeducation text. One self-compassion exercise involving listening to an audio.
8	Strengthening the components of self-compassion in university and everyday life	Psychoeducation	One psychoeducation text.

Module 1 – This module aims to teach college students about psychological distress, highlighting how these symptoms are associated with academic life stressors such as pressure for results and deadlines, competitiveness among classmates, separation from family, and beliefs about academic performance. It introduces the role of self-compassion as an emotional self-regulation mechanism. Additionally, it provides guidelines on using the app, including how the app works, the length of exercises, and how to prepare the personal environment for using the app.

Module 2 – This module introduces the self-judgment component. The authors considered it more viable to start with self-judgment because it is a common issue among college students. The module discusses the reasons behind self-criticism, such as beliefs about academic performance and self-efficacy. Module 2 highlights the importance of self-compassion in mitigating the harmful effects of self-criticism and teaches exercises to reduce self-criticism.

Module 3 – Module 3, as it addresses the component of self-kindness, highlights the importance of being kind, understanding and tolerant of oneself in the face of difficulties that arise in university life, such as not getting an internship or performing insufficiently on a task. Module 2 teaches two exercises for practicing self-kindness.

Module 4 – This module addresses the importance of mindfulness in university life and provides mindfulness exercises focusing on self-compassion. It is more practical than the previous modules. The reason it was presented in the middle of the intervention is that the researchers believed it was initially more appropriate to introduce the components of “self-judgment × self-kindness,” as this prior knowledge would facilitate the understanding of mindfulness in self-compassion.

Module 5 – This module addresses the common humanity component. Essentially, it emphasizes the importance of college students understanding that their perceived difficulties and suffering are shared experiences that other students also face. The exercises taught in this module aim to reinforce the sense of common humanity.

Module 6 – This module extends the discussion from Module 5, emphasizing the potential harmful effects of isolation. It proposes exercises designed to help college students feel less isolated while also encouraging self-kindness.

Module 7 – The seventh module addresses the remaining dimension of self-compassion, which is overidentification. This module highlights the importance of learning to recognize the thoughts and emotions that may occur in university life without neglecting or overestimating them. It teaches another mindfulness practice to cultivate this skill and is more practical compared to the other modules.

Module 8 – The last module provides a brief summary of the key points of the intervention and encourages college students to practice self-compassion on a daily basis to deal with the suffering arising from university life.

**Figure 1:**
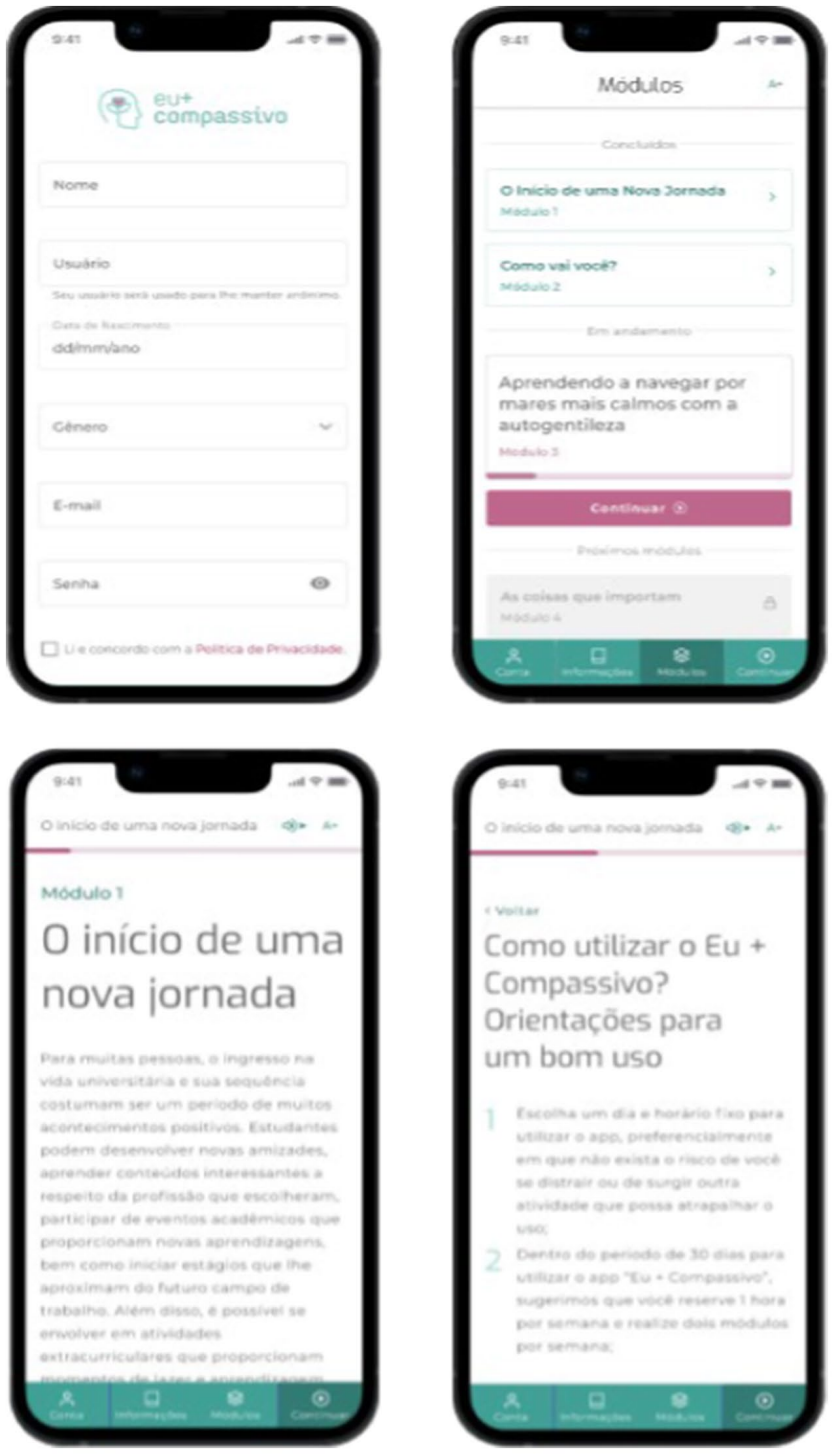
Screens of Eu + Compassivo.

The app was developed in the JavaScript language and was based on principles of the user-centered approach. The app prioritizes features such as anonymity, confidentiality, and data security. It is currently accessible on both Android and iOS platforms. A prior study highlighted excellent usability and acceptability of the tool among Brazilian college students and informatic workers (Schaab et al., 2024). Figure 1 presents some screenshots of the app.

### 2.2 Design

This pilot study used a pre-experimental design, involving a single group with both pre-test and post-test assessments (Shaughnessy et al., 2015). We assessed the intervention results using mixed methods (Creswell and Clark, 2011). The study protocol was previously registered on the Brazilian Clinical Trials platform^1^.

### 2.3 Participants

Initially, 99 undergraduate college students were recruited, surpassing the minimum requirement of 18 participants suggested by the sample calculation (Viechtbauer et al., 2015) and the minimum of 12 participants recommended in the scientific literature (Julious, 2005). The participants constituted a non-probabilistic and convenience sample (Shaughnessy et al., 2015). To be eligible, students had to declare at least mild symptoms of anxiety, stress, or depression due to issues in university life (as indicated through qualitative feedback from potential participants to researchers), be enrolled in an undergraduate course at a Brazilian university, possess a smartphone with Android and internet access, and declare proficiency in using a smartphone.

The participants’ mean age was 27.53 years (SD = 8.55 years). The majority of students originated from the southern region of Brazil (85.9%) and were enrolled in a public university (94.9%). Regarding smartphone usage patterns, 97% of participants reported using their smartphone more than 1 h daily, while 84.8% reported routinely using more than 3 apps on their cell phones. A significant portion of participants were in psychotherapy in parallel with the study (51.5%), and 47.5% were utilizing psychotropic medicines. Detailed demographic data are presented in Table 2.

**Table 2:** Sample characteristics.

Variable	*n*	%
Sex		
Male	28	28.3
Female	71	71.7
Region of Brazil		
South	85	85.9
Southeast	6	6.1
Midwest	2	2
Northeast	5	5.1
North	1	1
University		
Public	94	94.9
Private	5	5.1
Stage of Course		
First years (1st–4th semester)	53	53.5
Final years (5th–12th semester)	44	44.4
Above 12th semester	2	2
Daily cell phone usage time		
Less than 1 h	3	3
Among 1 h and 5 h	49	49.5
Among 6 h and 10 h	34	34.3
Among 11 h and 15 h	10	10.1
More than 16 h	3	3
Number of apps used daily		
Among 1 and 3	15	15.2
Among 4 and 6	38	38.4
Among 7 and 9	20	20.2
More than 10	26	26.3
Being in psychotherapy		
Yes	51	51.5
No	48	48.5
Using psychotropic medicine		
Yes	47	47.5
No	52	52.5

### 2.4 Measurements and instruments

#### 2.4.1 Mental health outcomes

##### 2.4.1.1 Primary outcomes

Depression, Anxiety, and Stress – 21– item Depression, Anxiety and Stress Scale (DASS-21): Originally developed by Lovibond and Lovibond (1995), the DASS-21 assesses symptoms of depression, anxiety, and stress. Responses to its items are recorded on a Likert scale ranging from 0 to 3 (“strongly disagree” to “strongly agree,” respectively). In Brazil, adaptation and validation for the adult population were conducted by Vignola and Tucci (2014), demonstrating satisfactory internal consistency, with Cronbach’s alpha values of 0.92 for the depression subscale, 0.86 for anxiety, and 0.90 for stress. To obtain the score for each dimension, the sum of all items should be calculated and then multiplied by 2, resulting in a score ranging from 0 to 42.

Emotional well-being – WHO-5: this generic measure evaluates emotional well-being over the preceding 14 days (Topp et al., 2015). Comprising five items rated on a Likert scale from 0 to 5, it yields a score ranging from 0 to 25. Higher scores indicate greater emotional well-being. The WHO-5 exhibits satisfactory reliability.

Self-compassion – Self-Compassion Scale: originally proposed by Neff (2003) and validated in Brazil by Souza and Hutz (2016), this scale comprises six factors: self-kindness, self-judgment, mindfulness, over-identification, common humanity, and isolation. With 26 items scored on a Likert scale from “almost never” (1) to “almost always” (5). The study reported a Cronbach’s alpha value of 0.92 for the overall scale and values ranging between 0.66 and 0.81 for each factor, indicating adherence to the original factorial structure and confirming the instrument’s validity. After reversing the punctuation of specific items, the score for each dimension is determined by calculating the average of all its items. The overall self-compassion score is then computed as the average across self-kindness, self-judgment, mindfulness, over-identification, common humanity, and isolation.

Compassion – Santa Clara Brief Compassion Scale: developed by Hwang et al. (2008), this unidimensional scale aims to succinctly measure compassion with five items on a Likert scale ranging from “not at all true for me” (1) to “very true for me” (7). In validation among the Brazilian population, the scale demonstrated satisfactory internal consistency, with a Cronbach’s alpha of 0.84 (Marchetti et al., 2018). Each item should be summed, resulting in a total compassion score ranging from 5 to 35.

Emotion Regulation – Emotional Dysregulation Scale – Adults (EDEA): we employed a condensed version of the Emotional Self-Regulation Scale (EARE). Comprising 15 items rated on a 4-point Likert scale, with endpoints labeled as “none of the times/not at all” (0) and “always” (3) (Cremasco et al., 2020), this scale encompasses four factors: adequate coping strategies, externalization of aggression, pessimism, and paralysis. Validation in Brazil demonstrated consistent internal validity, with values ranging from 0.68 to 0.96 for each factor (Cremasco et al., 2020). The score for each subscale is the sum of its respective items. The total score for the subscales of adequate coping strategies, pessimism, and paralysis ranges from 0 to 12, while for externalizing aggression, it varies between 0 and 9. Higher scores indicate increased emotion dysregulation.

##### 2.4.1.2 Secondary outcomes

Positive and Negative Affects – Positive and Negative Affect Schedule (PANAS): PANAS is a succinct two-dimensional self-report measure that assesses negative and positive affects (Watson et al., 1988). With 10 items in each affective dimension, respondents evaluate their experiences using a Likert scale that ranges from “not at all” (1) to “extremely” (5). In its Brazilian adult version, the instrument underwent validation and adaptation by Zanon et al. (2013), exhibiting satisfactory Cronbach’s alpha values. The total scores for negative affect and positive affect range from 5 to 50 and are obtained by summing the 10 items in each construct. Higher scores indicate greater intensity of the respective affects.

Satisfaction with Life – Life Satisfaction Scale (LSS): developed by Diener et al. (1985), this unidimensional measure assesses the cognitive facet of subjective well-being. LSS consists of 5 items on a Likert scale with anchors 1 and 7 denoting “strongly disagree” and “strongly agree.” Validated in Brazil by Zanon et al. (2014), LSS exhibited evidence of validity and satisfactory Cronbach’s alpha. The sum of item scores yields a total score between 5 and 35, with higher values indicating greater life satisfaction.

Hope – Cognitive Hope Scale (CHS): originating from Staats (1989), this measure focuses on the cognitive dimension of hope rooted in the Beckian theory of depression. Comprising 21 items arranged in a two-dimensional structure (self-centered hope and altruistic hope), responses are recorded on a two-column 5-point Likert scale. The scale captures both the desire and expectation aspects for each item (0 = do not want to; 5 = really want to; 0 = do not believe; 5 = believe a lot). In Brazil, the Cognitive Hope Scale underwent adaptation and validation by Pacico and Bastianello (2014) and demonstrated Cronbach’s alpha values of 0.86 for self-centered hope and 0.80 for altruistic hope. Expectation and desire scores for each item must be multiplied. The total cognitive hope score is the sum of these multiplied items, resulting in a score between 0 and 525. A higher total score indicates a higher level of cognitive hope.

Optimism – Life Orientation Test-Revised (LOT-R): developed by Scheier et al. (1994), this instrument assesses dispositional optimism through a unidimensional structure. Comprising 10 items, with 3 measuring optimism, 3 measuring pessimism, and 4 serving as “filter” items whose scores are not computed (Bastianello et al., 2014), responses are scored on a scale from 1 to 5, with anchors “strongly disagree” and “strongly agree.” The total scale score results from adding its six items, with three negative items being inverted. The LOT-R provides a total score between 6 and 30, with a higher score indicating a greater level of optimism. In Brazil, the LOT-R underwent validation and adaptation by Bastianello et al. (2014), demonstrating internal validity and a Cronbach’s alpha value of 0.80.

#### 2.4.2 Other outcomes

Adherence – Determined by the ratio between the scheduled intervention modules and those effectively achieved by the participants.

Satisfaction – Intervention Satisfaction: an *ad hoc* measure was devised to evaluate satisfaction and acceptability of the intervention. This instrument comprises 6 items, assessed on a Likert-type scale ranging from 1 to 10 (“strongly disagree” and “strongly agree”).

Acceptability and Impressions Regarding the Intervention – Intervention and App Impressions and Perceptions Questionnaire: a qualitative questionnaire was formulated to encompass impressions, perceptions and acceptability regarding both the intervention and the app.

### 2.5 Procedures

Firstly, the study was shared on the authors’ social networks and within public and private universities in Brazil. Prospective participants expressed their interest by reaching out to the study’s main author (BLS) through email or WhatsApp. Upon contact, they received comprehensive information about the research procedures and made a voluntary decision to participate. All students who consented to join the study were placed on a waiting list for approximately 3 weeks to ensure simultaneous initiation of app usage.

Seven days prior to starting using the app, participants were provided with the Consent Form (*CF*) and the pre-test form through Google Forms. All participants provided their consent by clicking on option “yes, I have read the terms and agree to participate in the research” before responding to research forms. Subsequently, participants received a link for accessing the Play Store, allowing them to download and utilize the app on their personal smartphones. The app usage occurred within a natural context, granting students the flexibility to choose the day, time, and location for engaging in the activities that best suited their routines. Participants were advised to use the app for a minimum period of 30 days, completing two modules per week, with an additional 15 days provided to accommodate any potential interruptions to the intervention.

**Figure 2:**
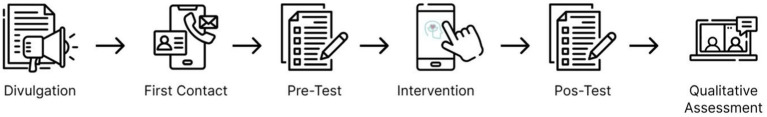
Procedures steps.

Seven days later, after completion of the intervention, participants were provided with the post-test questionnaire and a satisfaction and acceptability measure, administered through a Google Forms survey. Also, all participants provided their consent by clicking on option “yes, I have read the terms and agree to participate in the research.” Additionally, they were invited to respond to qualitative questions regarding their experience with the app. This step was facilitated by a trained research (DCS) assistant using either Google Meet or WhatsApp, based on the participant’s preference. Figure 2 displays a comprehensive view of the research procedures.

### 2.6 Data analysis procedures

All quantitative and categorical data were analyzed in the Statistical Package for the Social Sciences (SPSS), version 28.0 (SPSS^®^ Inc., Chicago, IL, United States). Quantitative sociodemographic data, outcomes, and cross-sectional measures of the intervention were summarized using mean, minimum, and maximum values, along with standard deviation. Categorical data were summarized using frequency.

Initially, the data normality was verified using the Shapiro–Wilk test (Field, 2017), which suggested a normal distribution. Therefore, the paired Student’s *T*-test (Field, 2017) was employed with a *p-*value of <0.05 in order to evaluate the benefits of the intervention. Effect sizes for each assessed outcome were computed using Cohen’s *d*. For interpreting effect sizes, Cohen’s (1998) guidelines were considered: scores between 0 and 0.2 indicate a null effect size; scores between 0.2 and 0.5 indicate a small effect size; scores between 0.5 and 0.8 indicate a medium effect size; finally, scores above 0.8 indicate a large effect size.

Qualitative data were analyzed using Thematic Analysis (Braun and Clarke, 2006). Initially, the audios from interviews were transcribed into text format, creating a document in docx format. Two researchers (BLS and DCS) independently reviewed this content and identified themes, adhering strictly to guidelines proposed for Braun and Clarke (2006). We adopted a data-driven approach, abstaining from *a priori* categories and theories in the interpretation of the qualitative data (Braun and Clarke, 2022). The steps undertaken included: (1) data familiarization, comprising repetitive and in-depth reading of qualitative data; (2) generation of initial codes, involving the identification of meaningful cores capable of generating themes; (3) theme identification, wherein these codes evolve into broader themes; (4) themes review, verifying the accurate representation of the qualitative database by the identified themes; (5) themes defining, which also involves assessing the presence of sub-themes. After these steps, the two authors met to deliberate and define the final thematic.

### 2.7 Ethical procedures

This research rigorously adhered to all ethical procedures as outlined in the Declaration of Helsinki for research involving human beings. Prior to commencing the study, approval was obtained from the Research Ethics Committee of the Federal University of Health Sciences of Porto Alegre (CAAE: 43804621.8.0000.5345). All participants provided their consent both in pre-test and post-test by reading the consent form and choosing the option “yes, I have read the terms and agree to participate in the research.”

## 3 Results

### 3.1 Adherence to the intervention

Since 99 participants were expected to utilize each of eight modules of the app, a total of 792 app usage modules were anticipated. Three hundred of the total 792 intervention modules were achieved, representing a 37.87% overall adherence. Approximately 68.69% of participants finished at least one module, with 26.26% completing all eight modules. The number of participants decreased by 50% between the first and fourth modules but remained relatively stable from the fifth to the eighth (11.35% decrease). Module four took the longest time (*M* = 1116.06 s; SD = 992.49 s) to be finished, while module eight was completed the fastest (*M* = 370.77 s; SD = 183.43 s). Table 3 provides an overview of the intervention’s adherence metrics.

**Table 3:** Adherence to intervention.

Module	Number of completions (%)	Minimum time (seconds)	Maximum time (seconds)	Mean time (SD)
Module 1	68 (68.69%)	83	1922	613.24 (313.18)
Module 2	51 (51.51%)	264	4,050	1087.06 (642.52)
Module 3	39 (39.39%)	178	3,087	1059.18 (688.04)
Module 4	34 (34.34%)	92	5,250	1116.06 (992.49)
Module 5	29 (30.29%)	84	2014	782.21 (490.06)
Module 6	27 (27.27%)	192	1895	610.59 (440.98)
Module 7	26 (26.26%)	82	2,452	639.54 (551.52)
Module 8	26 (26.26%)	60	861	370.77 (183.43)

SD, standard deviation.

### 3.2 Benefits of the intervention

Only participants who responded to both the pre-test and post-test and completed all eight modules of the intervention were included in the analysis of the app’s benefits. Thus, the benefits of the intervention were analyzed using data from 23 out of the initial 99 participants. Improvements in mental health scores were observed across all 21 assessed outcomes, as the reduction of psychopathological symptoms (e.g., depression) or the enhancement of positive mental health (e.g., positive affects). In 15 of these outcomes, the results were statistically significant, demonstrating a small, medium or large effect size. Anxiety (*d* = 0.52), stress (*d* = 0.62), negative affects (*d* = 0.66), and pessimism (*d* = 0.77) exhibited a decrease in scores with a medium effect size. Conversely, self-compassion demonstrated improvement with a large effect size (*d* = 1.15). Additionally, there was a large effect size increase in self-kindness (*d* = 0.95), common humanity (*d* = 1.05), and mindfulness scores (*d* = 0.94), accompanied by a large effect size decrease in self-judgment (*d* = 0.85), isolation (*d* = 0.97), and a medium effect size in over-identification (*d* = 0.76) scores. Positive affects (*d* = 0.45), optimism (*d* = 0.44) and life satisfaction (*d* = 0.48) also exhibited score improvements with a small effect size. Finally, emotional well-being exhibited score improvement with a medium effect size (*d* = 0.54). Table 4 provides a comprehensive presentation of intervention’s benefits.

**Table 4:** Benefits of app on mental health indicators (*n* = 23).

Outcome	Instrument	Pre-test	Post-test	*T*(*p*)	Effect size
Primary outcomes					
Depression	DASS-21	16.52 (10.13)	11.74 (9.19)	1.99 (0.059)	0.42
Anxiety	DASS-21	10.78 (9.57)	5.39 (6.62)	2.50 (0.020)*	0.52
Stress	DASS-21	17.83 (9.16)	10.87 (7.86)	2.98 (0.007)**	0.62
Emotional well-being	WHO-5	10.17 (4.75)	13 (5.03)	−2.59 (0.017)*	0.54
Compassion	SCBCS	26.17 (7.02)	27.57 (5.24)	−1.56 (0.133)	0.33
Appropriate Coping Strategies	EDEA	6.00 (2.70)	5.00 (2.86)	1.88 (0.072)	0.39
Externalization of Aggression	EDEA	1.3 (1.22)	0.87 (1.06)	1.73 (0.96)	0.36
Pessimism	EDEA	6.39 (3.22)	4.35 (2.46)	3.69 (0.001)*	0.77
Paralyzation	EDEA	7.48 (3.20)	6.30 (3.32)	1.70 (0.103)	0.35
Self-compassion	SCS	2.34 (0.64)	3.09 (0.74)	−5.50 (0.000)**	1.15
Self-kindness	SCS	2.39 (0.80)	3.12(0.92)	−4.54 (0.000)**	0.95
Self-judgment	SCS	3.88 (0.92)	3.08 (0.88)	4.09 (0.000)**	0.85
Common humanity	SCS	2.43(0.78)	3.30(0.92)	−5.05 (0.000)**	1.05
Isolation	SCS	3.85 (0.80)	2.98 (1.02)	4.67 (0.000)**	0.97
Mindfulness	SCS	2.78 (0.96)	3.46(0.92)	−4.50 (0.000)**	0.94
Over-identification	SCS	3.77 (0.85)	3.23 (0.86)	3.63 (0.001)**	0.76
Secondary outcomes					
Hope	CHS	310.35 (96.17)	327.13 (101.20)	−1.62 (0.119)	0.34
Negative affect	PANAS	28.47 (8.50)	22.57 (10.28)	3.14 (0.005)**	0.66
Positive affect	PANAS	28.04 (7.81)	30.95 (9.80)	−2.16 (0.042)*	0.45
Life satisfaction	SWLS	19.65 (6.75)	21.57 (7.48)	−2.28 (0.033)*	0.48
Optimism	LOT-R	19.73 (5.13)	21.30 (6.03)	−2.10 (0.047)*	0.44

*p < 0.05, **p < 0.01; d = T/√n.

### 3.3 Satisfaction and acceptability of the intervention

All six indicators assessing the satisfaction and acceptability of the intervention had an average score above seven, with five indicators averaging above eight. The statement “I think the app can help with the emotional well-being of college students “received the highest rating (*M* = 8.87; SD = 1.42), while the statement “I will continue using the app if I do not feel good” received the lowest rating (*M* = 7.30; SD = 2.58). Table 5 presents a comprehensive overview of the global assessment of satisfaction.

**Table 5:** Measure of acceptability and satisfaction with the intervention (*n* = 23).

Item	Minimum	Maximum	Mean (SD)
1. The app is relevant	5	10	8.26 (1.60)
2. I felt satisfied using the app	4	10	8.17 (1.87)
3. I would recommend other people to use the app	5	10	8.81 (1.56)
4. I think the app can help with the emotional well-being of college students	5	10	8.87 (1.42)
5. I think the app was able to help me	4	10	8.04 (1.85)
6. I will continue using the app if I do not feel good	1	10	7.30 (2.58)

### 3.4 Qualitative assessment

Nineteen participants agreed to participate in the qualitative research step. The qualitative analysis revealed two main themes. They are, respectively, named “perceptions about the app and the intervention,” and “perceptions of personal benefits.” The theme perceptions about the app and the intervention captures feedback patterns concerning the format and presentation of the app. It comprises four subthemes: ease of use, reflecting on the app’s user-friendliness; intervention acceptance, indicating participants’ receptiveness to the intervention; length of the texts, suggesting participants’ perceptions of extensive textual content; and organization of the routine, pointing to potential barriers in executing the intervention, such as the demands of classes, internships or work.

The theme perceptions of personal benefits address the perceived advantages by participants, categorized into three subthemes: self-knowledge, demonstrating that the app may have contributed to participants’ enhanced self-awareness; emotional well-being, focusing on the perceived mental health benefits of the app; and self-compassion, indicating participants’ acquired self-compassion skills. Table 6 presents these themes, subthemes, and some excerpts exemplifying the analyses.

**Table 6:** Thematic analysis.

Thematics	Subthematics	Excerpts
1. 1. Perceptions about the app and the intervention	1.1. Ease of use	“No difficulties regarding the app. None at all. Very easy to use.” (Man, 24 years) “I managed to do all the modules, exercises too. I thought they were well explained. I could understand everything correctly and it was very smooth. Very easy to use.” (Woman, 20 years old) “The app itself is very easy. I found it very easy to understand. The texts were very explanatory.” (Man, 28 years old)
	1.2. Intervention acceptance	“I liked it very much. I liked it so much that I recommended it to friends at university. There was a friend who already came to give me feedback and she really liked it. I really liked the app.” (Woman, 31 years old) “My experience was good, I liked it. It was very interesting to have an app that I think I had been looking for some time. So, participating in this project, this experience, was really cool. Having an app in the palm of your hand that helps was very interesting, I liked it, I really liked it.” (Woman, 26 years old) “I loved it. I loved using the app. I loved it. If I had my way, the app would have 30 modules to use one per day. It was literally really, really cool to use.” (Man, 24 years old)
	1.3 Length of the texts	“I think a weak point, which was actually important for me, is that I saw that there was a lot, a lot, a lot of text. I, for example, get a little lost with texts.” (Woman, 22 years old) “[…] A little time passed, and I actually did not want to read that text. I put it on to listen. Then I found it a little boring. Just read and do the activities… I found it a little boring, but overall, I liked it.” (Woman, 24 years old) “I would, I guess, include less written part (texts).” (Woman, 22 years old)
	1.4 Organization of the routine	“In my case, I’m working and studying, so I had little time. But a student who is just studying… He has to study, for sure, but he’ll have a little more time to finish the 8 modules.” (Man, 24 years old) “The only difficulty I had was maintaining the rhythm of using the app on the same days of the week, at the same time.” (Woman, 20 years old)
2. Perceptions of personal benefits	2.1. Self-knowledge	“I think I came away with a vision, a different perspective on my relationship with myself, you know? I did not just take it to university life, but also other aspects of life, other areas.” (Woman, 22 years old) “I was very open to questions about the app. And I ended up being a little more open with myself because of that. Because, anyway, I had some questions that I had never stopped to think about. I had to stop, reflect, see how I was acting towards myself, what I acted differently towards others to be able to respond.” (Woman, 25 years old)
	2.2 Emotional well-being	“I think this app for mental health was very useful, because when I was using it I was in the middle of the semester, from the middle to the end. So, it was a time when I was anxious about handing in assignments and exams. Every week when I practiced, I was able to relax, have less anxiety and think about things in a different way, from a perspective where I would not be so stressed.” (Woman, 22 years old) “There were times when I was really stressed about college. Then I read those texts and they were saying ‘oh I know you are stressed […] everyone goes through this’. That’s exactly what I was feeling. So, I really enjoyed using the app, as there were those moments, which kind of was exactly what I needed. I really liked it.” (Woman, 18 years old) “I think the app really helped me. It felt like I was talking to someone. That part was positive. Almost like a consultation with a psychologist.” (Woman, 28 years old)
	2.3 Self-compassion	“Sometimes I would push myself and then review what I had learned on the app and have a more understanding look at myself. So, I think the app helped me a lot with that. Understanding that I also have limits. That sometimes our maximum is not satisfactory, but the fact that we are giving our best is something that we have to understand that this is our limit for that moment, for that day.” (Woman, 31 years old) “I see that there was help (from the app), from the moment I was able to understand that I’m not the only one going through these difficulties. I was able to open up, talk to other people about it. I started to be more understanding with myself, understanding that, in the same way that others have difficulties, I also have it and it’s not just them who have good things.” (Woman, 25 years old) “(The app) gave me some insights into punishing myself less. Regarding when things go wrong.” (Man, 43 years old)

## 4 Discussion

Before conducting randomized clinical trials, which may demand considerable time, financial funds, and high specialized staff, pilot studies are recommended to assess key variables that may affect the success of these interventions (Lancaster, 2015; Lowe, 2019). Consequently, methodological guidelines propose the prior evaluation of indicators, such as the initial benefits of the intervention, its acceptability, interest, and participant’s satisfaction (Lancaster, 2015; Teresi et al., 2022). The evidence assessed in pilot studies facilitates adjustments in the presentation, format, and active ingredients of interventions (Schaab and Remor, 2023). This adjustment aims to enhance personalization and attractiveness, ultimately increasing the likelihood of achieving effectiveness in the targeted outcomes (Lowe, 2019; Schaab and Remor, 2023). In line with these considerations, this pilot study assessed the adherence to the intervention, its preliminary benefits, user satisfaction, acceptability, and general qualitative impressions regarding the app. Each of these topics is expounded upon in the subsequent sections.

### 4.1 Adherence to intervention

The final intervention adherence rate of 26.26% aligns with scores identified in digital intervention studies in natural contexts addressing psychological distress. Evidence suggests that only between 0.5 and 28.6% of users complete interventions or persist in using them after 6 weeks (Fleming et al., 2018). Regarding mindfulness apps, the user retention rate is just 4.7% after 30 days of use (Baumel et al., 2019). The observed trend of a gradual decrease in app usage or module completion over time in the present study is also consistent with findings in similar research (i.e., Fleming et al., 2018; Baumel et al., 2019; Linardon and Fuller-Tyszkiewicz, 2020). Several factors are associated with the decline in app usage in natural contexts, including the absence of immediate benefits, competition with other smartphone apps, and a lack of user motivation and engagement (Giebel et al., 2023).

Regarding barriers to completing the modules, we formulated two hypotheses: the module size and the mental health status of individuals in the sample. Initially, we believe that the length of texts and information in app may have impacted adherence to the intervention. Some users qualitatively suggested that engaging in the intervention involved reading or listening to an extensive amount of information, and this could be a barrier. Evidence on adherence to digital interventions indeed suggests that information overload can pose a potential barrier to intervention adherence (Giebel et al., 2023). Therefore, it is important that interventions are also attractive, especially to people experiencing depressive symptoms.

It is plausible that the mental health status of the sample is associated with adherence to the app modules. Individuals with higher levels of depressive symptoms tend to exhibit lower engagement in app-based interventions (Zeng et al., 2020; Molloy and Anderson, 2021), as well as in face-to-face psychological services and psychological resources programs (Zeng et al., 2020). This behavior may be attributed to the depressive symptoms themselves, which encompass disinterest, lack of energy, motivation, tiredness, and apathy (Molloy and Anderson, 2021). Given that the majority of the sample in this research manifested severe symptoms of depression and anxiety, it is conceivable that these symptoms influenced adherence.

In the future, gamification could be a strategy to increase user adherence and retention rates, as it has been associated with better engagement rates in app-based interventions (Jakob et al., 2022). Furthermore, another promising strategy is the creation of a forum within the app. Users could anonymously share their experiences with self-compassion practices, making the experience of using the app more collective and even more attractive, since several app-based interventions have adopted social media elements as a way to become more attractive (Wei et al., 2020).

For the implementation of the clinical trial, we will consider a potential sample attrition of approximately 75% by the conclusion of the intervention. To enhance engagement and adherence, an augmented strategy will involve the implementation of more reminders and weekly notifications for participants. These reminders aim to encourage continued utilization of the app.

### 4.2 Intervention benefits

The intervention results indicate that the app may be effective in promoting the mental health of Brazilian college students, demonstrating a reduction in psychopathological symptoms and an improvement in positive mental health indicators. These findings align with evidence from interventions conducted in face-to-face settings, which have also shown medium effect sizes in decreasing self-judgment (Wakelin et al., 2022), anxiety and depression (Kirby et al., 2017), and enhancing self-compassion, mindfulness and subjective well-being (Kirby et al., 2017).

The identified benefits are consistent with findings from two known digital self-compassion interventions targeting college students (Andersson et al., 2021; Serlachius et al., 2021). The Whitu app designed for New Zealand university students demonstrated effectiveness in decreasing anxiety and stress, while enhancing subjective well-being over a six-week period, with observed medium effect sizes (Serlachius et al., 2021). Similarly, a digital intervention grounded in self-compassion principles, delivered to Swedish university students, proved effective, exhibiting a large effect size in stress reduction and self-compassion improvement within the experimental group as compared to the control group (Andersson et al., 2021).

Regarding self-compassion interventions among college students delivered in a face-to-face format and online format, the present research presented a higher effect size (*d* = 1.15) than the synthesis of other studies (*g* = 0.49) (Póka et al., 2024). Also, the effect size of self-compassion in the present study (*d* = 1.15) is similar to that of the synthesis of face-to-face interventions (*g* = 0.80) (Póka et al., 2024). However, it is important to highlight that the results of behavioral interventions may vary depending on the characteristics of the audience, psychological instruments, and, mainly, the intervention itself, including psychological techniques, experience and previous training of facilitators, and intervention length.

Studies employing structural equation modeling have underscored the association between self-compassion and mental health. An investigation assessing the advantages of a guided Positive Psychology intervention delivered via email for adult mental health, using a mediation model, revealed that self-compassion emerged as the most proficient construct in elucidating the decrease in anxiety and depression and the enhancement of emotional well-being (Schotanus-Dijkstra et al., 2019). Similarly, an eight-week mindfulness-based intervention demonstrated that self-compassion mediated the direct effects of the intervention on stress (Sevel et al., 2020). These mediation effects provide insights into how the app may have positively influenced Brazilian college students.

Self-compassion and self-judgment yielded the most substantial effect sizes in the current study. The improvement in self-compassion is likely associated with a decrease in psychological distress, along with improvements in emotion regulation dimensions, such as pessimism. Given that emotion regulation serves as a mediator in the connection between self-compassion and mental health (Fong and Loi, 2016), it is plausible that college students utilized learned self-compassion strategies to manage the intensity and frequency of negative emotions linked to university life. A central emphasis of the intervention was to introduce and cultivate a set of self-compassionate skills specifically helpful to academic stressors, with a primary focus on fostering self-kindness, cultivating a sense of common humanity, and practicing mindfulness.

It is important to note that the development of the app strictly adhered to the self-compassion literature, which contributes to the positive outcomes of the study. The level of compassion content in psychological interventions is linked to its enhancement (Jazaieri et al., 2013). Thus, before creating the prototype, psychologists specializing in Positive Psychology reviewed the intervention to ensure that the content and techniques used were scientifically appropriate and could be effective among college students. A recent qualitative review evidenced on Android and iOS platforms 24 apps to promote compassion, but only nine of which are properly based on scientific evidence (Krijger et al., 2023), which reinforce the importance of solid theoretical foundations.

The observed benefits in the current study are promising and indicate the feasibility of conducting a randomized clinical trial in the near future. The incorporation of a control group, randomization, and follow-up procedures will enable a comprehensive assessment of the app’s effectiveness, ensuring both robust evaluation and the examination of result consistency over time.

Finally, it is important to note that the results cannot yet be generalized, primarily due to the study’s sample size and the fact that it was only conducted in Brazil. However, the positive results obtained, especially in the self-compassion components, encourage further studies to evaluate the benefits of practicing self-compassion among university students, particularly in Latin America. Most studies of app-based self-compassion interventions are currently concentrated in Europe.

### 4.3 Intervention’s satisfaction

All satisfaction indicators for the intervention were positive, with scores consistently exceeding the established cutoff of 7 in previous pilot studies (Schaab and Remor, 2023). The satisfaction observed is likely linked to perceived benefits, as participants recognized the potential advantages for both them and other college students. This satisfaction is highlighted in responses to the item, “I think the app can help with the emotional well-being of college students,” which garnered the highest average score.

Conversely, the item “I will continue using the app if I do not feel good” received the lowest rating. This lower score can be attributed to the fact that some users, upon completing the intervention, may not be inclined to revisit the same activities. Consequently, implementing app modifications becomes crucial to sustain ongoing user interest in the tool.

Additionally, it is noteworthy that there is a substantial score variation between each item, ranging from 1 to 10 in certain instances and, more commonly, between 5 and 10. These findings indicate that a minority of participants expressed dissatisfaction with the intervention. Such discrepancies were expected, considering individual preferences regarding the content and format of the app.

In summary, the satisfaction measure indicated favorable outcomes for the app. However, it is essential to acknowledge that the satisfaction assessment was conducted solely among the 23 participants who completed the entire intervention. This potential bias could have influenced the positive evaluation of the intervention. Next, it is crucial to gather data from participants who did not complete all the modules to discern whether the dropout is associated with the app itself or arises from personal issues.

### 4.4 Acceptability and qualitative impressions

The qualitative data suggests the acceptance of the app by participants, as confirmed through the identified subthemes “ease of use” and “mental health.” It was expected that participants would encounter no difficulties on app use, which aligns with a previous study that indicated the excellent usability of Eu + Compassivo. Furthermore, earlier data on participants’ smartphone use revealed that almost 97% of them spent more than an hour a day on their cell phones and 84% used more than 3 apps, highlighting their digital literacy. The widespread use of mobile technologies among university students positions them as an opportune audience for the administration of digital interventions (Kern et al., 2018).

Conversely, it is crucial to acknowledge that, for at least some participants, the volume of text in the intervention was a potential barrier. Usability research involving other mental health apps has indicated a preference for more direct information presented in a playful manner (Jessen et al., 2018). Future versions of the app will aim to modify the presentation format and the extent of information, especially because playful elements such as gamification have been recommended in the development of online interventions (Jakob et al., 2022). Furthermore, participants identified a challenge in integrating the use of the app into their study, internship, and work routine, underscoring the importance of a more concise and attractive intervention.

Also, it is crucial to highlight that studies’ participants perceived benefits from using the app on their mental health aligning with the quantitative data. The specific enhancement in emotional well-being may stem from acquiring self-compassion strategies for emotion regulation and self-knowledge, as suggested by these subthemes. As reflected in the results, many participants appear to have broadened their sense of shared humanity, a component of self-compassion, acknowledging that other college students also experience suffering, which means they are not alone. Additionally, some university college students reported a reduced tendency for self-criticism, aligning with the self-judgment dimension of self-compassion.

The evidence regarding personal benefits aligns with findings from other digital self-compassion interventions. A study qualitatively assessed the effects of the Self-Compassion app on British university students (Beaumont et al., 2022). After 6 weeks of use, students reported an increased ability to feel and practice self-compassion in their daily lives.

In summary, the present evidence indicates that app is accepted, easy to use, and can provide benefits to the emotional well-being of college students. Changes to the format and presentation of the app may be necessary in the future in order to make its content more succinct and objective.

### 4.5 Limitations

The first limitation is the significant dropout in the intervention. Although dropout rates are in line with app-based intervention studies in natural contexts, the small number of participants who completed the intervention and responded to the pre-test and post-test (*n* = 23) suggests caution in interpreting the benefits of the intervention.

The second limitation of the study pertains to the substantial number of participants concurrently engaging in psychotherapy or utilizing psychotropic drugs while utilizing the app, particularly antidepressants and benzodiazepines. While this introduces a confounding variable, it is crucial to acknowledge that both the average duration of psychotherapy and the usage of psychopharmaceuticals exceed 3 years, potentially mitigating the impact of these variables.

Finally, the last potential limitation is the absence of a control group. Although control groups are not deemed obligatory in pilot studies, the inclusion of a control group could have aided in mitigating experimental biases.

### 4.6 Strengths

To the best of our knowledge, our app stands as one of the initial app-based intervention based on self-compassion principles designed to enhance the mental well-being of college students worldwide. Additionally, in the context of Brazil, digital interventions based on self-compassion are not currently prevalent, which highlights the innovative nature of our study. Conducting this research in a natural setting allows us to comprehend the anticipated user engagement and real-world benefits of the app.

## 5 Conclusion

The results of this pilot study suggest that the app may be beneficial for Brazilian college students and is accepted and well-received by at least those who have completed all intervention. However, despite adherence rates falling within the expected range for studies conducted in natural contexts, there is a need for activities to enhance intervention adherence, such as weekly usage reminders and increased dynamism in content presentation and exercises. The next step involves conducting a randomized clinical trial, comparing participants using this Brazilian self-compassion app with those on a waiting list. We expect still in 2024 to distribute the app free of charge among Brazilian college students facing mental health impairments.

## Data Availability

The dataset generated during the current study are not publicly available due nature of the information provided by the research participants. Requests to access the dataset should be directed to the corresponding author BS, bruno.schaab@ufcspa.edu.br.

## Footnotes

^1^https://ensaiosclinicos.gov.br/rg/RBR-5ghn5f4
